# Highly expressed B3GALT5‐AS1 contributes to gastric cancer progression by recruiting WDR5 to mediate B3GALT5 and regulating β‐catenin/ZEB1 axis

**DOI:** 10.1111/jcmm.70061

**Published:** 2024-09-03

**Authors:** Wei Feng, Yelan Tang, Rongrong Jing, Shaoqing Ju, Wei Zong

**Affiliations:** ^1^ Department of Laboratory Medicine Affiliated Hospital of Nantong University Nantong Jiangsu China; ^2^ Medical School of Nantong University Nantong University Nantong Jiangsu China; ^3^ Research Center of Clinical Medicine Affiliated Hospital of Nantong University Nantong Jiangsu China

**Keywords:** B3GALT5, B3GALT5‐AS1, gastric cancer, metastasis, WDR5

## Abstract

Long non‐coding RNAs (lncRNAs) play an important role in the progression of gastric cancer (GC), but its specific regulatory mechanism remains to be further studied. We previously identified that lncRNA B3GALT5‐AS1 was upregulated in GC serum. Here, we investigated the functions and molecular mechanisms of B3GALT5‐AS1 in GC tumorigenesis. qRT‐PCR was used to detect B3GALT5‐AS1 expression in GC. EdU, CCK‐8, and colony assays were utilized to assess the proliferation ability of B3GAL5‐AS1, and transwell, tube formation assay were used to assess the invasion and metastasis ability. Mechanically, FISH and nuclear plasmolysis PCR identified the subcellular localization of B3GALT5‐AS1. RIP and CHIP assays were used to analyse the regulation of B3GALT5‐AS1 and B3GALT5. We observed that B3GALT5‐AS1 was highly expressed in GC, and silencing B3GALT5‐AS1 could inhibit the proliferation, invasion, and migratory capacities of GC. Additionally, B3GALT5‐AS1 was bound to WDR5 and modulated the expression of B3GALT5 via regulating the ZEB1/β‐catenin pathway. High‐expressed B3AGLT5‐AS1 promoted GC tumorigenesis and regulated B3GALT5 expression via recruiting WDR5. Our study is expected to provide a new idea for clinical diagnosis and treatment.

## 1 INTRODUCTION

Gastric cancer (GC) is one of the most common malignant tumours worldwide. According to a statistical report on cancer in 2023, GC ranks fifth in the incidence and mortality of gastrointestinal tumours.^1^ Although the incidence of GC has decreased in recent years, chronic H. pylori infection is the most powerful pathogenic factor of GC in China.^2^ As a result, the incidence of GC remains high in our country. At the same time, due to the lack of sensitive and specific diagnostic markers for GC, most of the patients were found in the late stage, and extensive invasion and metastasis have become one of the main causes of death from GC.^3^, ^4^, ^5^ However, the exact aetiology and mechanisms of GC remain unclear.

Long non‐coding RNAs (lncRNAs) can be divided into five categories according to their positions related to coding genes in the genome.^6^ Among them, antisense lncRNAs are a kind of lncRNAs transcribed by antisense chains of genes and overlap with the mRNA of this gene.^7^ It has been reported that approximately 70% of genes have antisense lncRNAs, and antisense lncRNAs can participate in the pathophysiological process of diseases by regulating the expression of endogenous genes of the positive‐sense strand.^8^, ^9^ Previously, we screened lncRNA β‐1,3‐galactosyltransferase5‐AS1 (B3GALT5‐AS1) via GC microassay and literature reference^10^, and our study confirmed that B3GALT5‐AS1 was upregulated in the serum of GC patients.^11^ However, the mechanisms underlying the oncogenic functions of B3GALT5‐AS1 require further investigation. In addition, according to the different localization of antisense lncRNAs, their regulatory mechanisms for homologous genes are diverse. lncRNAs located in the cytoplasm can directly bind to and stabilize homologous sense genes. Meanwhile, antisense lncRNAs can form different splicing variants that have opposite effects on related genes. lncRNAs located in the nucleus can interact with chromatin regulatory proteins to promote their recruitment to chromatin, thereby regulating transcriptional activity and affecting the expression of adjacent related genes.^12^, ^13^, ^14^ B3GALT5 is a homologous sense gene of B3GALT5‐AS1 and has been reported to be associated with cancer progression and can serve as a cancer biomarker for diagnosis.^15^, ^16^ In this study, we aimed to delineate the clinical significance of B3GALT5‐AS1 and investigate its involvement in GC progression by regulating B3GALT5.

## 2 MATERIALS AND METHODS

### 2.1 GC tissue samples

All GC tissue samples were collected from patients who underwent surgery at the Affiliated Hospital of Nantong University (Nantong, China) between June 2018 and July 2020. Imaging and postoperative pathological reports indicated GC in all patients, who were not treated with chemotherapy or other drugs and had no other digestive diseases. This study was approved by the Clinical Research Ethics Committee of the Affiliated Hospital of Nantong University (Ethical Review Report No. 2021‐L048).

### 2.2 Cell lines and culture

Human GC cell lines (SGC‐7901, AGS, MKN‐45, MGC‐823 and HGC‐27) and a gastric epithelial cell line (GES‐1) were purchased from the Chinese Academy of Sciences Committee on Type Culture Collection Cell Bank (Shanghai, China). GES‐1 and GC cells were attached to the wall in a complete culture medium at 37°C in a 5% CO_2_ cell incubator. The complete medium consisted of RPMI‐1640/DMEM (Corning, USA) supplemented with 10% fetal bovine serum (FBS).

### 2.3 RNA extraction and quantitative real‐time polymerase chain reaction (qRT‐PCR)

Trizol Reagent (Invitrogen, Carlsbad, CA, USA) was used to extract total RNA. The concentration and purity of RNA were determined using a micro‐spectrophotometer (Thermo Scientific, USA). Two microgram total RNA reversed transcription into complementary DNA (cDNA) using the Revert Aid First Strand cDNA Synthesis Kit (Thermo Scientific, MA, USA). The expression of B3GLAT5‐AS1 was determined by qRT‐PCR using SYBR Green Master Mix (Roche, Basel, Switzerland) according to the manufacturer's protocol. 18S rRNA was used as an internal control. The expression levels of these genes were calculated by the 2^−ΔΔCt^ method. Specific primer sequences used in this study are listed in Table S1.

### 2.4 Cell transfection

B3GALT5‐AS1 short hairpin RNAs (shRNA) and pcDNA‐B3GALT5‐AS1 were obtained from GenePharma (Suzhou, China). B3GALT5 and WDR5 small interfering RNA (siRNAs) were purchased from RiboBio (Guangzhou, China). Transfection was performed using Lipofectamine 3000 (Invitrogen, Carlsbad, Calif, USA), according to the manufacturer's instructions. The shRNA and siRNA sequences used for the transfection are listed in Table S2.

### 2.5 Cell proliferation assays

Cell proliferation ability was detected by using EdU cell proliferation, Cell Counting Kit‐8 (CCK‐8), and colony formation assay. The cell‐light EdU Apollo567 In Vitro Kit (RiboBio, Guangzhou, China) was used to detect cell proliferation. In total of, 40,000 transfected cells were grown in 24‐well plates, incubated with EdU, fixed with 4% paraformaldehyde and incubated with Apollo567. Stained cells were photographed using a fluorescence microscope.

The CCK‐8 assay kit (Dojindo, Kumamoto, Japan) was used to measure cell viability. In total of, 3000 transfected cells were grown in 96‐well plates and incubated with 10 μL CCK‐8 solution for 10 min, and absorbance was measured at 450 nm every 24 h. All the experiments were repeated five times.

The transfected cells were collected, the suspended cells were diluted, 600 cells per well were added to a six‐well plate, and 10% was replenished to 2 mL. Culture was in the box for 14 days, during which the fresh medium was changed every 4 days. The culture was terminated when the cells were distributed in clumps under the microscope, forming visible patches of white cells. After washing with PBS, 0.1% crystal violet dye solution was added for staining and counting.

### 2.6 Flow cytometry assay

After 48 h of transfected with sh‐B3GALT5‐AS1/pcDNA‐B3GALT5‐AS1 and the negative control, GC cells were collected and cell cycle assays were performed according to the manufacturer's protocol.

### 2.7 Cell migration and invasion assays

A wound healing assay was used to detect the cell migration ability. The 48 h transfected cells were placed on a 6‐well plate, and after the cells were attached to the wall and grown on the full plate, a cross line was crossed with a 10 μL tip. The scratch distance was photographed under a microscope and the distances between 0 and 24 h were compared.

The transfected cells of each group were collected, and the cell density was adjusted to (1–10) × 10^5^/mL. The transwell chamber was placed in a 24‐well plate and 600 μL medium containing 20% FBS was added to the lower chamber. For the invasion assay, cells were added to the upper chamber pre‐coated with Matrigel (BD Bioscience). Three random fields per chamber were counted using a microscope (Olympus). All experiments were repeated three times.

### 2.8 Tube formation assay

GC cell culture medium transfected with B3GALT5‐AS1 related vector for 48 h was co‐cultured with HUVECs. After 6 h of culture, the ability of HUVECs to form tubules was observed and photographed under an inverted microscope.

### 2.9 Tumour xenografts in null mice

Five‐week‐old male nude mice were divided into two groups, MKN‐45 cells were injected subcutaneously (transfected with NC plasmid or B3GALT5‐AS1‐sh1 or B3GAL5‐AS1‐sh2,1 × 10^7^/mL, 200 μL). The length, width, and height of the xenograft tumours were measured every 3 days. After 30 days, the tumours were excised and weighed. The xenograft tumours were cut and subjected to immunohistochemical staining.

### 2.10 Subcellular location of B3GLAT5‐AS1 and B3GALT5

FISH and subcellular fractionation analyses were used to determine the distribution of B3GALT5‐AS1 and B3GALT5. Cytoplasmic RNA and nuclear RNA were extracted using a nuclear‐cytoplasmic separation kit. qRT‐PCR was used to detect relative expression. U6 was used as the nuclear control, and GAPDH was used as the cytoplasmic control. The pretreated cells were first added with probe pre‐hybridization solution and incubated for 30 min at 37°C, then the probe containing the designed B3GALT5‐AS1 and B3GALT5 and incubated overnight at 37°C with hybridization solution. Finally, cells were stained with DNA and observed under a fluorescence microscope.

### 2.11 Pull‐down analysis

The biotin‐labelled B3GALT5‐AS1 probe was designed in advance and tested using the operation steps of Pierce™ Magnetic RNA‐Protein Pull‐Down Kit (Thermo Scientific, Rockford, lL, USA). Finally, the obtained protein bands were silver stained, and specific bands were selected for mass spectrometry analysis.

### 2.12 Qualitative protein analysis (Shotgun)

Shotgun LC–MS/MS mass spectrometry to identify the protein mixture. Protein strips were digested with protease into peptide mixtures, which were then separated by HPLC and imported in series into a high‐resolution mass spectrometer for analysis. After the peptide is ionized in the mass spectrometer, it will carry a certain amount of charge. Through the detector analysis, the mass‐to‐charge ratio (*m*/z) of each peptide can be obtained. At the same time, the mass spectrometer will further bombard the peptide ions to obtain the secondary mass spectrum signal. The mass spectrometry data were analysed using the retrieval software and the corresponding proteome database.

### 2.13 RNA immunoprecipitation (RIP) assay

Cells were treated with the RIP kit (BersinBio, Guangzhou, China) according to the manufacturer's instructions. Samples with anti‐WDR5 and IgG antibodies were used to extract total RNA using the TRIzol reagent. The RNA samples were reverse‐transcribed according to the reverse transcription kit steps, and PCR was performed using the PCR system. The amplification effect of the final product was tested by 3% agarose gel electrophoresis.

### 2.14 Chromatin immunoprecipitation (ChIP) assay

Following the CHIP kit instructions, SGC‐7901 cells were fixed with 4% paraformaldehyde and incubated with glycinate for 10 min to produce DNA‐protein crosslinks. The cells were then lysed using cell lysis buffers, nuclear lysis buffers and ultrasound treatment to produce chromatin fragments of 200–300 bp. Next, lysate‐coupled magnetic protein A beads were immunoprecipitated. IgG was used as the negative control. Finally, PCR probes containing binding sites were designed to analyse the DNA precipitated by qRT‐PCR.

### 2.15 Western blotting analysis

The cells were lysed, protein samples were collected, total protein was extracted, and protein concentration was measured using the BCA method. After sample loading, electrophoresis, membrane transfer, sealing, primary antibody and secondary antibody incubation, ECL tablets were developed (Quantity One software, Bio‐Rad). Using GAPDH as the internal reference, ImageJ image analysis software was used to detect the expression of the relative proteins. The antibodies used are listed in Table S3.

### 2.16 Statistical analysis

All Data were analysed using SPSS software (version 20.0; IBM SPSS Statistics, Chicago, IL, USA) and GraphPad 9.0. Student's *t*‐test was conducted to analyse the differences in B3GALT5‐AS1 levels in GC tissues, one‐way analysis of variance (ANOVA) was used to analyse the different treated groups in our study, and a chi‐squared test was used for statistical analysis of clinicopathological features. *p* < 0.05.

## 3 RESULTS

### 3.1 Highly expressed B3GALT5‐AS1 is associated with poor prognosis in GC

**FIGURE 1:**
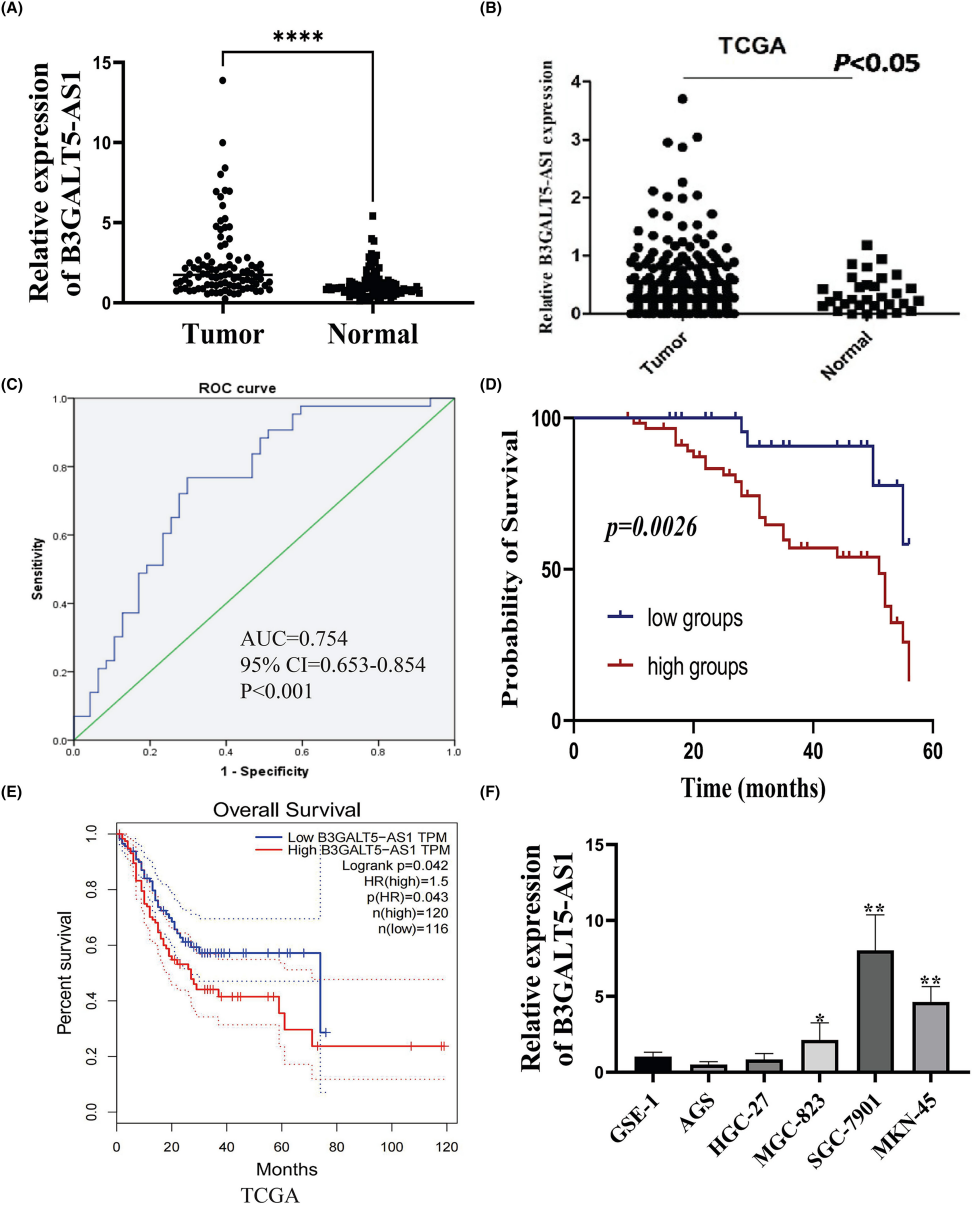
Highly expressed B3GALT5‐AS1 is associated with poor prognosis in GC. (A) B3GALT5‐AS1 expression in GC and adjacent normal tissues (*n* = 90). (B) Expression of B3GALT5‐AS1 from the TCGA database. (C) Diagnostic value of B3GALT5‐AS1. (D) Survival analysis comparing patients with high and low B3GALT5‐AS1 expression in GC using the Kaplan–Meier method. (E) Survival analysis downloaded from the TCGA database. (F) Expression of B3GALT5‐AS1 in GC cell lines (AGS, HGC‐27, BGC‐823, SGC‐7901 and MKN‐45) and the normal gastric epithelial cell line GES‐1. *p < 0.05, **p < 0.01, ***p < 0.001.

In our previous study, we confirmed that B3GLAT5‐AS1 was significantly increased in serum samples of GC patients and had potential auxiliary diagnostic value.^17^ Excitingly, we analysed the expression of BGALT5‐AS1 in 90 GC tissues and paired normal tissues and found that B3GALT5‐AS1 was upregulated in GC tissues compared with normal tissues (*p* < 0.05) (Figure 1A). Furthermore, according to the TCGA database, B3GALT5‐AS1 was also found to be highly expressed in GC, and highly expressed B3GALT5‐AS1 was associated with poor overall survival (Figure 1B,E). In addition, we also analysed the diagnostic value of B3GALT5‐AS1 in GC tissues, and the area under the receiver operating characteristic (ROC) curve was 0.754 (95% confidence interval [CI] = 0.653–0.854; *p* < 0.001), which indicated a better diagnostic efficiency (Figure 1C). Furthermore, we divided GC patients into two groups according to the mean value and analysed the correlation between B3GALT5‐AS1 expression and the clinical characteristics, and the results showed that the high expression of B3GALT5‐AS1 was significantly correlated with lymphatic metastasis and TNM stage (Table 1). Subsequently, we performed a survival prognosis analysis of these patients, and the results showed that the overall survival of patients with high B3GALT5‐AS1 expression was worse than that of patients with low B3GALT5‐AS1 expression (Figure 1D). qRT‐PCR was used to detect the expression of B3GALT5‐AS1 in the collected GC cell lines, and the results indicated that B3GALT5‐AS1 was upregulated in GC cell lines (Figure 1 F). These data suggest that B3GALT5‐AS1 expression was enhanced in GC, and the level of B3GALT5‐AS1 was significantly correlated with the survival prognosis of patients.

**TABLE 1:** The relationship between B3GALT5‐AS1 levels and clinicopathologic features of GC patients.

Characteristic	Total	B3GALT5‐AS1 (*n* %)		*χ*2 value	*p*
	*n*	Low *n* = 37	High *n* = 53		
Gender					
Male	52	22	30	0.082	0.775
Female	38	15	23		
Age (years)					
≤61	32	17	15	0.035	0.852
>61	58	20	38		
Tumour size (cm)					
≤5	54	23	31	1.755	0.261
>5	36	14	22		
Histological differentiation					
Well‐moderate	55	17	38	1.333	0.248
Poor	35	20	15		
TNM stage					
I and II	37	19	18	6.842	0.013^*^
III and IV	53	18	35		
Lymph node metastasis					
Yes	60	21	39	4.535	0.041^*^
No	30	16	14		

^*^ *p*<0.05

### 3.2 Knockdown of B3GALT5‐AS1 inhibits proliferation and cell growth in GC

**FIGURE 2:**
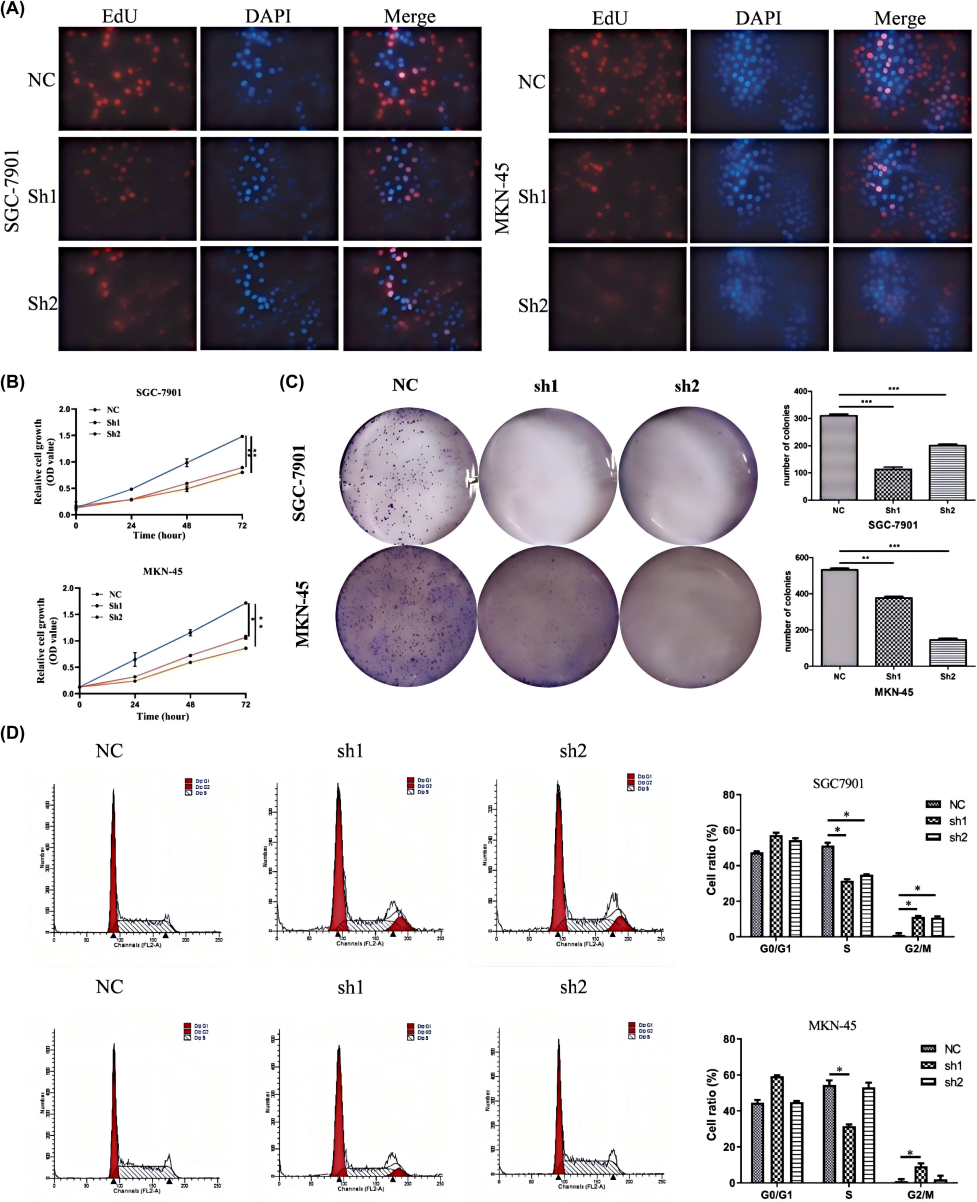
Highly expressed B3GALT5‐AS1 is associated with poor prognosis in GC. (A) EdU analysis showing the proliferation of SGC‐7901 and MKN‐45 cells transfected with sh‐B3GALT5‐AS1 or sh‐NC. (B) CCK‐8 assay showed proliferation abilities of B3GALT5‐AS1 (C) Colony formation assay was performed to determine the proliferation of SGC‐7901 and MKN‐45 cells transfected with sh‐B3GALT5‐AS1 or shNC. (D) Flow cytometry was used to determine the effect of sh‐B3GALT5‐AS1 on SGC‐7901 and MKN‐45 cell cycle. *p < 0.05, **p < 0.01, ***p < 0.001.

Based on these results, we selected SGC‐7901 and MKN‐45 cells for further study. We transfected the shRNA vector into these two cell lines, as well as an overexpression vector into AGS cell lines. qRT‐PCR was used to detect the knockdown efficiency and overexpression efficiency in GC cells (Figure S1). EdU cell proliferation, CCK‐8, and colony formation assays showed that silencing B3GALT5‐AS1 significantly reduced cell growth in vitro (Figure 2A–C). Flow cytometry was used to detect the cell cycle in GC cell lines. The results showed that knockdown of B3GALT5‐AS1 led to cell arrest in the G0/G1 phase and decreased in the S phase (Figure 2D). Conversely, overexpression of B3GALT5‐AS1 promoted cell proliferation, and the number of cells in the S phase increased, while there were no such changes in the control group (Figure S2A–D). All results corroborated that the knockdown of B3GALT5‐AS1 inhibited cell proliferation and contributed to cell cycle arrest in GC.

### 3.3 B3GALT5‐AS1 contributes to cell invasion, migration and angiopoiesis in GC

**FIGURE 3:**
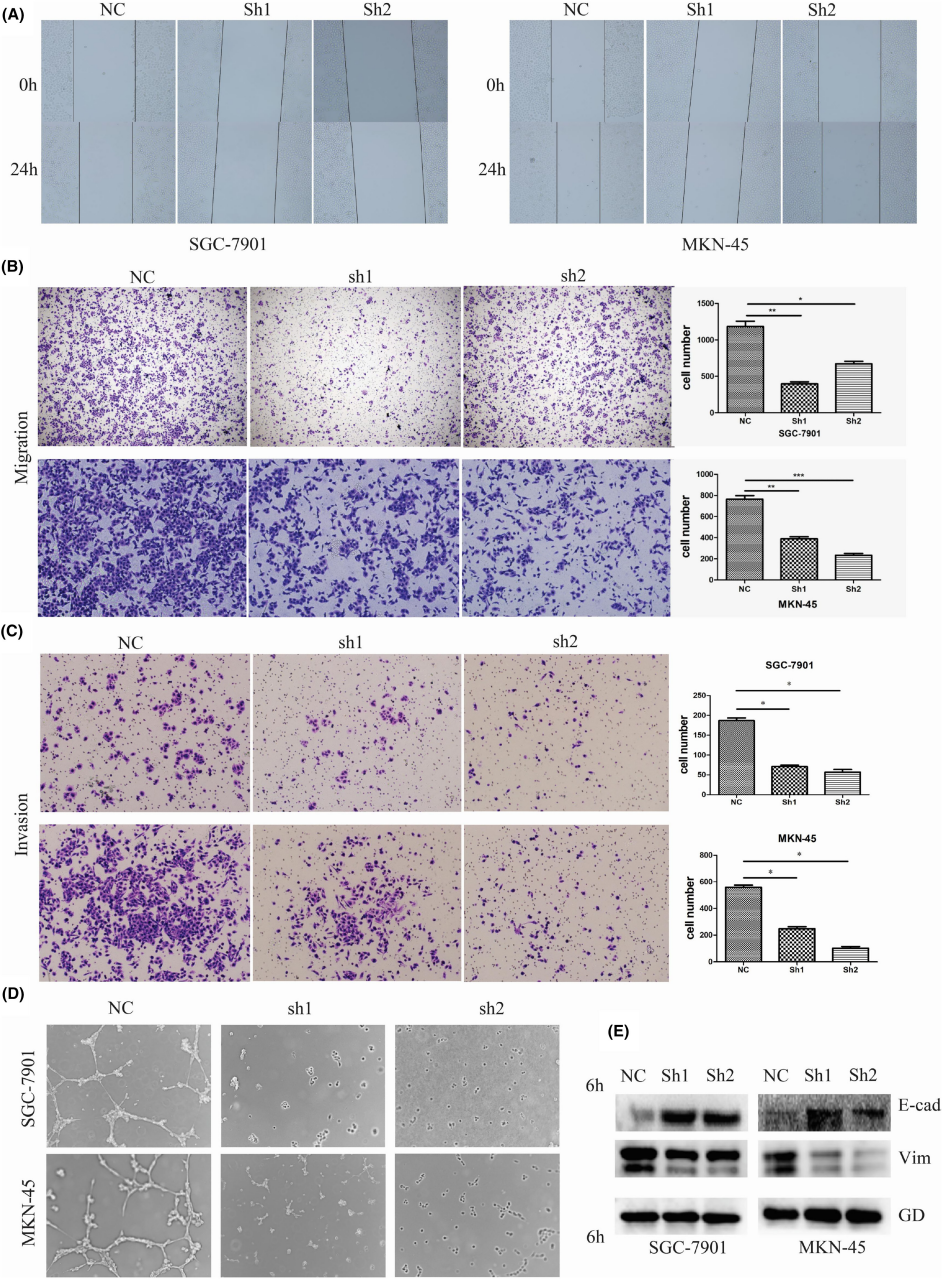
B3GALT5‐AS1 contributes to cell invasion, migration, and angiopoiesis in GC. (A) A wound healing assay was performed to detect the ability to knock down B3GALT5‐AS1. (B and C) Transwell assay was performed to measure the effect of B3GALT5‐AS1 knockdown on cell migration and invasion. (D) Tubule formation assay was performed to determine angiogenesis ability. (E) The influence of B3GALT5‐AS1 on EMT markers (E‐cadherin and Vimentin) in GC cells was measured using a western blot. *p < 0.05, **p < 0.01, ***p < 0.001.

Accumulating evidence has confirmed that extensive invasion and metastasis are the main causes of death in GC patients.^18^, ^19^ Moreover, the occurrence and development of GC is complicated and involves the dysregulation of multiple oncogenes and cancer suppressors, among which epithelial‐mesenchymal transition (EMT) and angiopoiesis play important roles in GC metastasis.^20^, ^21^, ^22^, ^23^ Subsequently, the wound healing assay revealed that silencing B3GALT5‐AS1 may markedly reduce GC cell migration (Figure 3A). The transwell migration assay also showed that B3GALT5‐AS1 knockdown significantly inhibited cell migration compared to the control groups in SGC‐7901 and MKN‐45 cells. In addition, transwell invasion assay analysis found that B3GALT5‐AS1 knockdown dramatically reduced the number of invaded SGC‐7901 and MKN‐45 cells in comparison with those in the control group (Figure 3B,C). In contrast, enhancing B3GALT5‐AS1 expression produced the opposite effect (Figure S2E,F). Moreover, the tube formation assay results showed that the tubule formation ability of HUVECs was decreased in GC cell culture medium with interference of B3GALT5‐AS1 (Figure 3D), while the tubule formation ability of HUVECs was enhanced in GC cell culture medium with overexpression of B3GALT5‐AS1 (Figure SG). Besides, the levels of EMT markers were also assessed in GC cells. As expected, interfering with B3GALT5‐AS1 could decrease the expression of interstitial marker Vimentin and increase the expression of epithelial marker E‐cadherin in GC cells (Figure 3E). Thus, we speculated that B3GALT5‐AS1 could affect the EMT process in GC. The above studies revealed that high expression of B3GALT5‐AS1 promoted GC cell invasion, migration, and angiogenesis, and facilitated tumour metastasis.

### 3.4 B3GALT5‐AS1 silencing inhibits tumour growth in vivo

**FIGURE 4:**
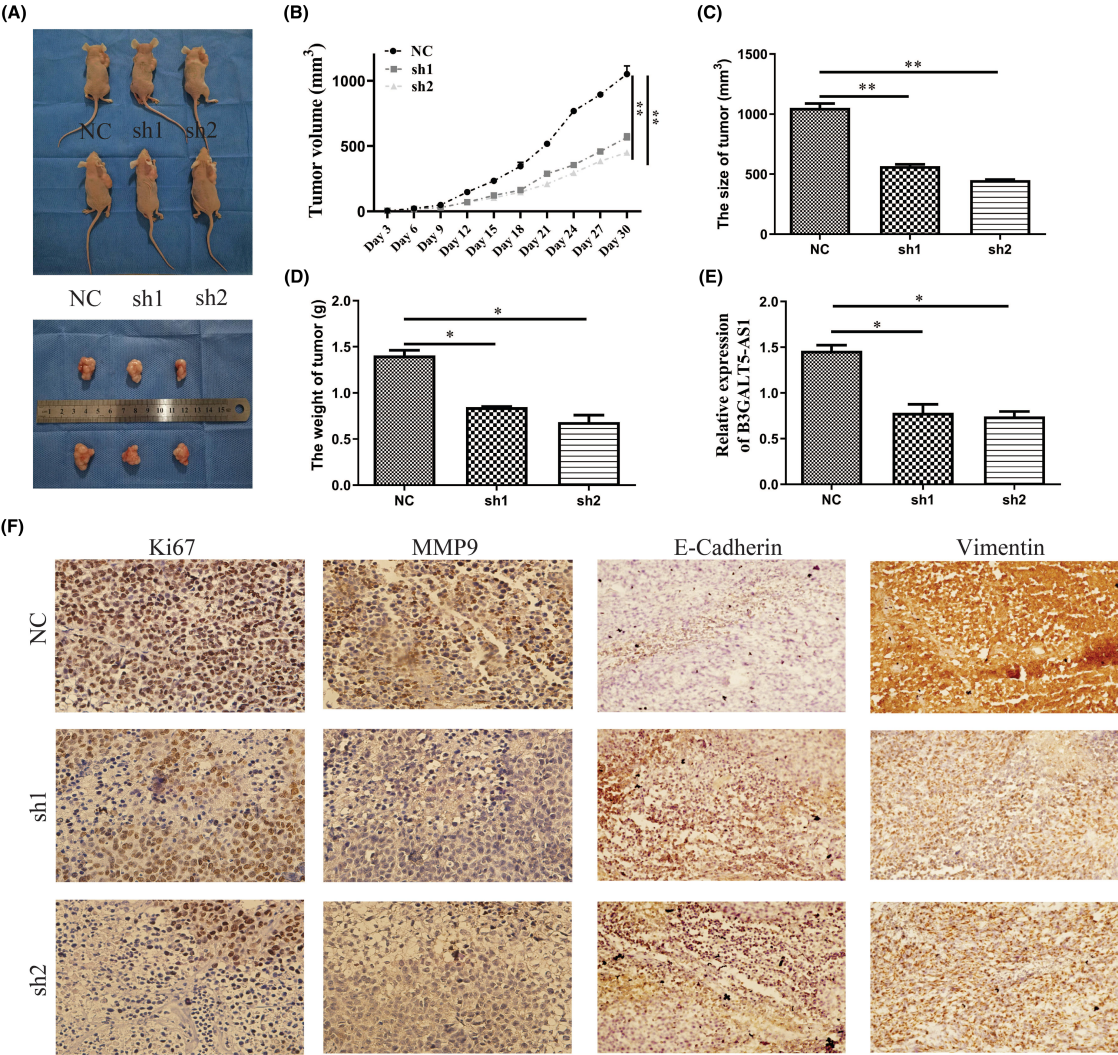
Knockdown of B3GALT5‐AS1 inhibited tumour growth in nude mice xenograft models. (A) Images of nude mice and transplanted tumours after 30 days of inoculation with NC and B3GALT5‐AS1‐sh1 or sh2‐treated MKN‐45 cells. (B) The tumour volume growth curve. (C) Tumour size. (D) Tumour weight. E: Relative expression by qRT‐PCR in the different groups. (E) Ki67, MMP9, E‐Cadherin, and Vimentin protein levels in tumour tissues from sh‐B3GALT5‐AS1 or negative control groups. *p < 0.05, **p < 0.01.

To further verify the effect of B3GALT‐AS1 on GC progression, we constructed a subcutaneous xenograft tumour nude mouse model for the following study. MKN‐45 cells (transfected with B3GALT5‐AS1‐sh1 or B3GAL5‐AS1‐sh2 or NC plasmid) were injected subcutaneously. After 30 days, the tumours from the knockdown group were remarkably smaller and developed more slowly than those in the control group (Figure 4A–D). Furthermore, qRT‐PCR analysis confirmed that B3GALT5‐AS1 expression in xenograft tumours in the knockdown group was lower than that in the control group (Figure 4E). In addition, immunohistochemical analysis showed that the expression levels of Ki67 and MMP9 in the sh‐B3GALT5‐AS1 group were lower than those in the control group. Meanwhile, to find out whether B3GALT5‐AS1 regulates EMT in vivo as well, we observed E‐Cadherin and Vimentin expression in GC tissues of nude mice by IHC. Compared to the control group, the B3GALT5‐AS1‐sh1 or sh2 group had upregulated E‐Cadherin and reduced Vimentin expression (Figure 4F). These results suggested that B3GALT5‐AS1 affects the proliferation and metastasis of GC cells in vivo.

### 3.5 B3GALT5‐AS1 modulates GC progression via B3GALT5/β‐catenin/ZEB1 pathway

**FIGURE 5:**
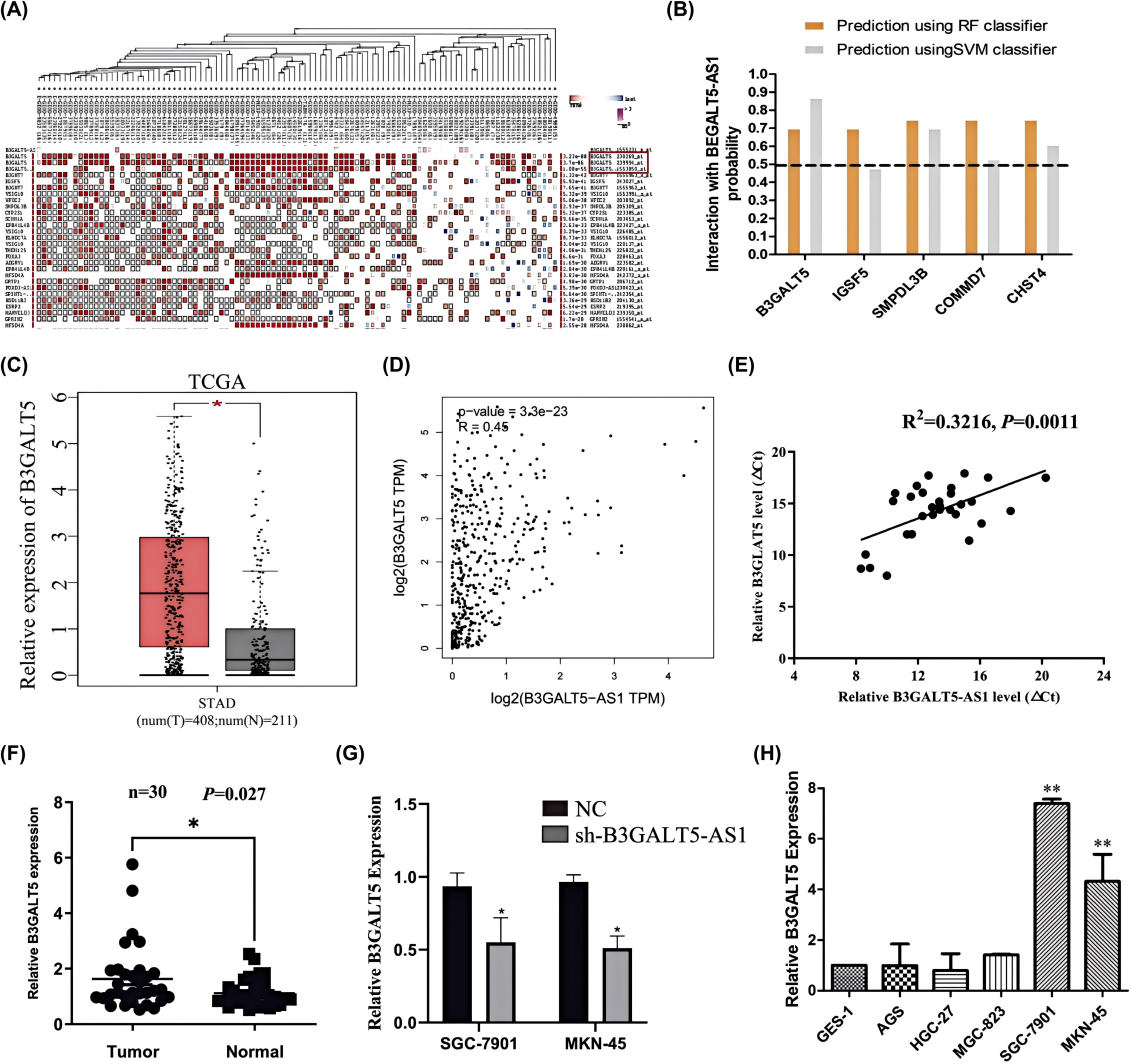
B3GALT5‐AS1 modulates GC progression via B3GALT5/β‐catenin /ZEB1 pathway. (A) Multi Experiment Matrix database screened the co‐expressed genes of lncRNA B3GALT5‐AS1. (B) RNA‐Protein Interaction Prediction confirmed the binding potential of co‐expressed genes. (C) Expression of B3GALT5 in TCGA GC cohort. (D) Correlation analysis of the expression of B3GALT5‐AS1 and B3GALT5 in the TCGA GC cohort. (E) Spearman‐Pearson correlation between B3GALT5‐AS1 and B3GALT5 expression in GC tissues. (F) QRT‐PCR analysis of the relative expression of B3GALT5 in GC tissues (*n* = 30). (G) QRT‐PCR was used to detect B3GALT5 expression after B3GALT5‐AS1 transfection. (H) Expression of B3GALT5 in GC cell lines. *p < 0.05, **p < 0.01.

To further verify the mechanism by which B3GALT5‐AS1 regulates the progression of GC, we downloaded the co‐expressed genes of lncRNA B3GALT5‐AS1 using the Multi Experiment Matrix database (MEM, https://biit.cs.ut.ee/mem/index.cgi). The results showed that its sense strand B3GALT5 had the highest correlation with lncRNA B3GALT5‐AS1 (Figure 5A). RNA‐protein interaction prediction (RPISeq) also confirmed this situation (Figure 5B). Accumulating evidence has verified that antisense lncRNAs can participate in the process of diseases by moderating the expression of endogenous genes of their sense genes.^9^ The data from the TCGA database showed that B3GALT5 was upregulated in GC tissues as well as B3GALT5‐AS1 (Figure 5C). GEPIA (Gene expression Profiling Interactive Analysis) Correlation analysis showed that B3GALT5‐AS1 expression was significantly positively correlated with B3GALT5 expression in GC tissues (Figure 5D). We also detected the relative expression of B3GALT5 in 30 GC tissues and cell lines via qRT‐PCR analysis. The results also showed that B3GALT5 was highly expressed in GC, and the expression of B3GALT5 was directly proportional to that of B3GALT5‐AS1 (Figure 5E,F). Furthermore, we found that the B3GALT5 level was also increased in GC cell lines. While silencing B3GALT5‐AS1 could significantly decrease the level of B3GALT5 (Figure 5G,H). The above studies confirmed that lncRNA B3GALT5‐AS1 has a significant correlation with its sense strand B3GALT5, and indicated that B3GALT5 is a potential target of lncRNA B3GALT5‐AS1.

**FIGURE 6:**
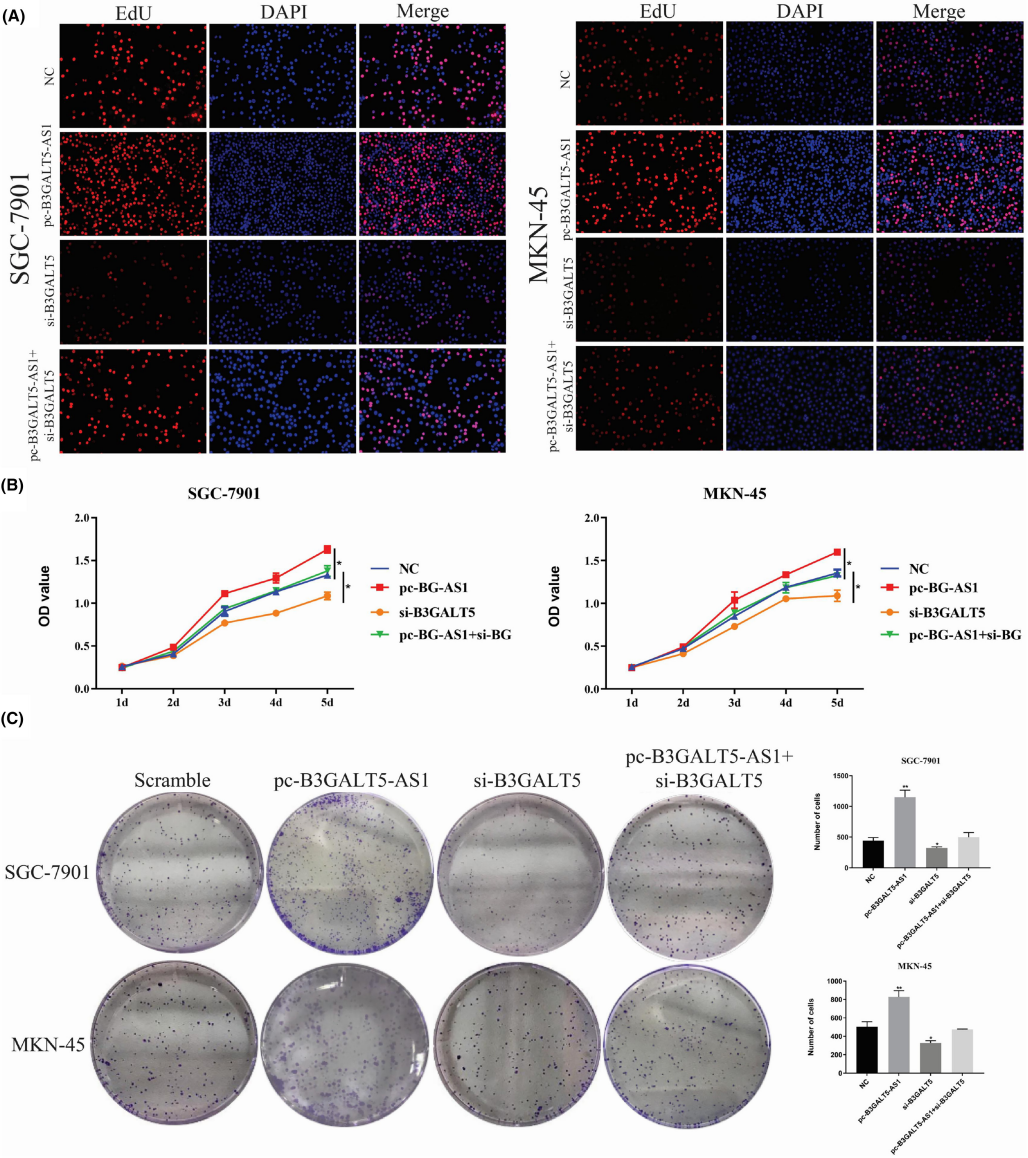
B3GALT5‐AS1 modulates GC progression via B3GALT5/β‐catenin/ZEB1 pathway. (A) EdU analysis showing the proliferation of SGC‐7901 and MKN‐45 cells transfected with pcDNA‐B3GALT5‐AS1, si‐B3GALT5, pcDNA‐B3GALT5‐AS1+si‐B3GALT5 or NC. (B) CCK‐8 assay showed proliferation abilities of SGC‐7901 and MKN‐45 cells transfected with pcDNA‐B3GALT5‐AS1, si‐B3GALT5, pcDNA‐B3GALT5‐AS1+si‐B3GALT5 or NC. (C) Colony formation assay was performed to determine the proliferation of SGC‐7901 and MKN‐45 cells transfected with pcDNA‐B3GALT5‐AS1, si‐B3GALT5, pcDNA‐B3GALT5‐AS1+si‐B3GALT5 or NC. *p < 0.05, **p < 0.01.

**FIGURE 7:**
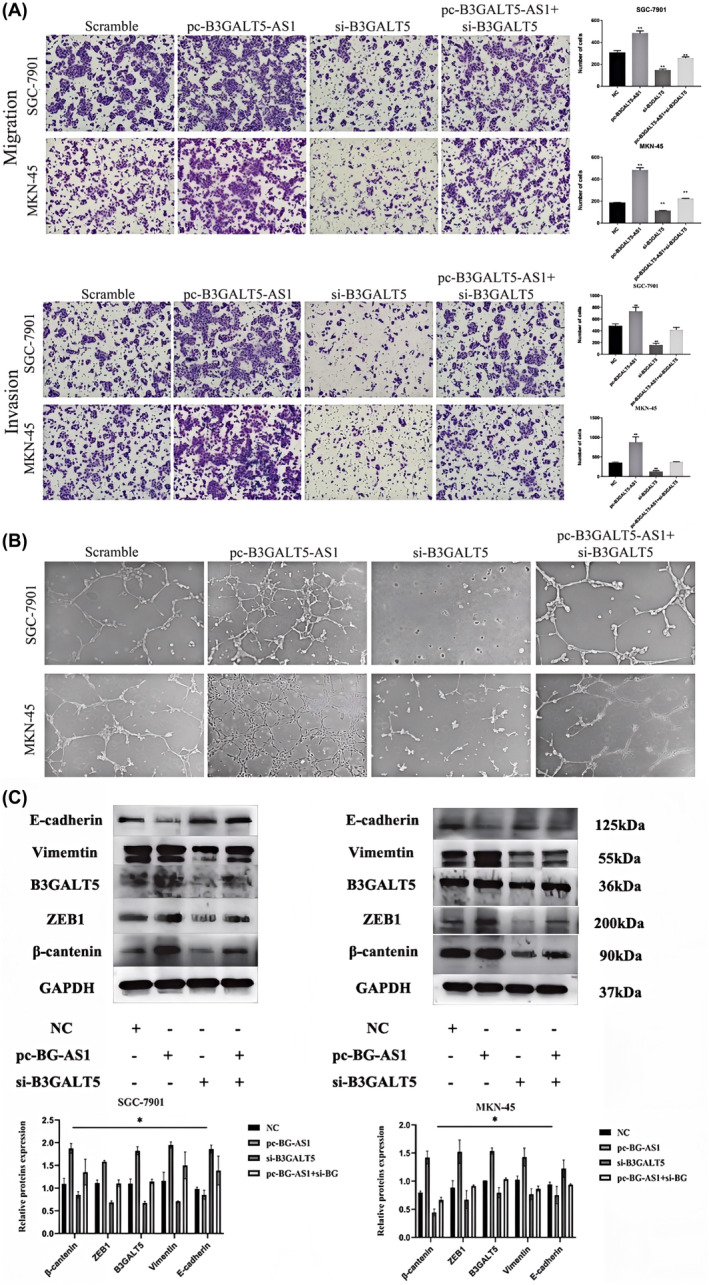
B3GALT5‐AS1 modulates GC progression via B3GALT5/β‐catenin /ZEB1 pathway. (A) Transwell assay was performed to measure the effect of transfecting with pcDNA‐B3GALT5‐AS1, si‐B3GALT5, pcDNA‐B3GALT5‐AS1 + si‐B3GALT5 or NC on cell migration and invasion. (B) Tubule formation assay was performed to determine angiogenesis ability. (C) The influence of transfected with pcDNA‐B3GALT5‐AS1, si‐B3GALT5, pcDNA‐B3GALT5‐AS1+si‐B3GALT5 or NC on EMT markers and β‐catenin /ZEB1 pathway markers in GC cells was measured using western blot. *p < 0.05, **p < 0.01.

To further verify the regulation of B3GALT5‐AS1 on B3GALT5, we performed rescue assays to validate whether B3GALT5 was involved in B3GALT5‐AS1 mediated regulation of GC progression. The results showed that the effects of si‐B3GALT5 on GC cell proliferation were partially reversed by overexpression of B3GALT5‐AS1 (Figure 6A–C). Similarly, the transwell assay and tube formation assay showed that silencing B3GALT5 may attenuate invasion and angiopoiesis of GC cells and that co‐transfection of pcDNA‐B3GALT5‐AS1 and si‐B3GALT5 could partly reverse the effect knockdown of B3GALT5 (Figure 7A,B). Studies have shown that B3GALT5 is upregulated in breast cancer and can regulate the expression of zinc finger E‐box binding homeobox transcription factor 1 (ZEB1) as well as the activation of β‐catenin nuclear translocation, promoting the progression of breast cancer metastasis.^16^ In our study, the highly expressed B3GALT5‐AS1 could promote the malignant progression of GC cells such as proliferation, invasion, and metastasis, while si‐B3GALT5 could partially inhibit the effects of B3GALT5‐AS1. Therefore, we hypothesized that B3GALT5 regulates GC EMT progression also by affecting the β‐catenin/ZEB1 axis. To further determine whether B3GALT5‐AS1 modulates GC metastasis via the EMT‐related pathway, we examined the expression of ZEB1 and β‐catenin, which are key factors in the activation of EMT.^24^, ^25^ Western blotting was used to detect these genes in different treatment groups. These observations suggested that after B3GALT5‐AS1 knockdown, E‐cadherin expression was upregulated, while B3GALT5, Vimentin, β‐catenin and ZEB1 expression were downregulated (Figure 7C). These results indicated that B3GALT5‐AS1 can indeed affect B3GALT5 level, and the differentially expressed B3GALT5 can regulate GC invasion and metastasis through the β‐catenin/ZEB1 axis.

### 3.6 B3GALT5‐AS1 positively regulates B3GALT5 expression in GC via recruiting WDR5

**FIGURE 8:**
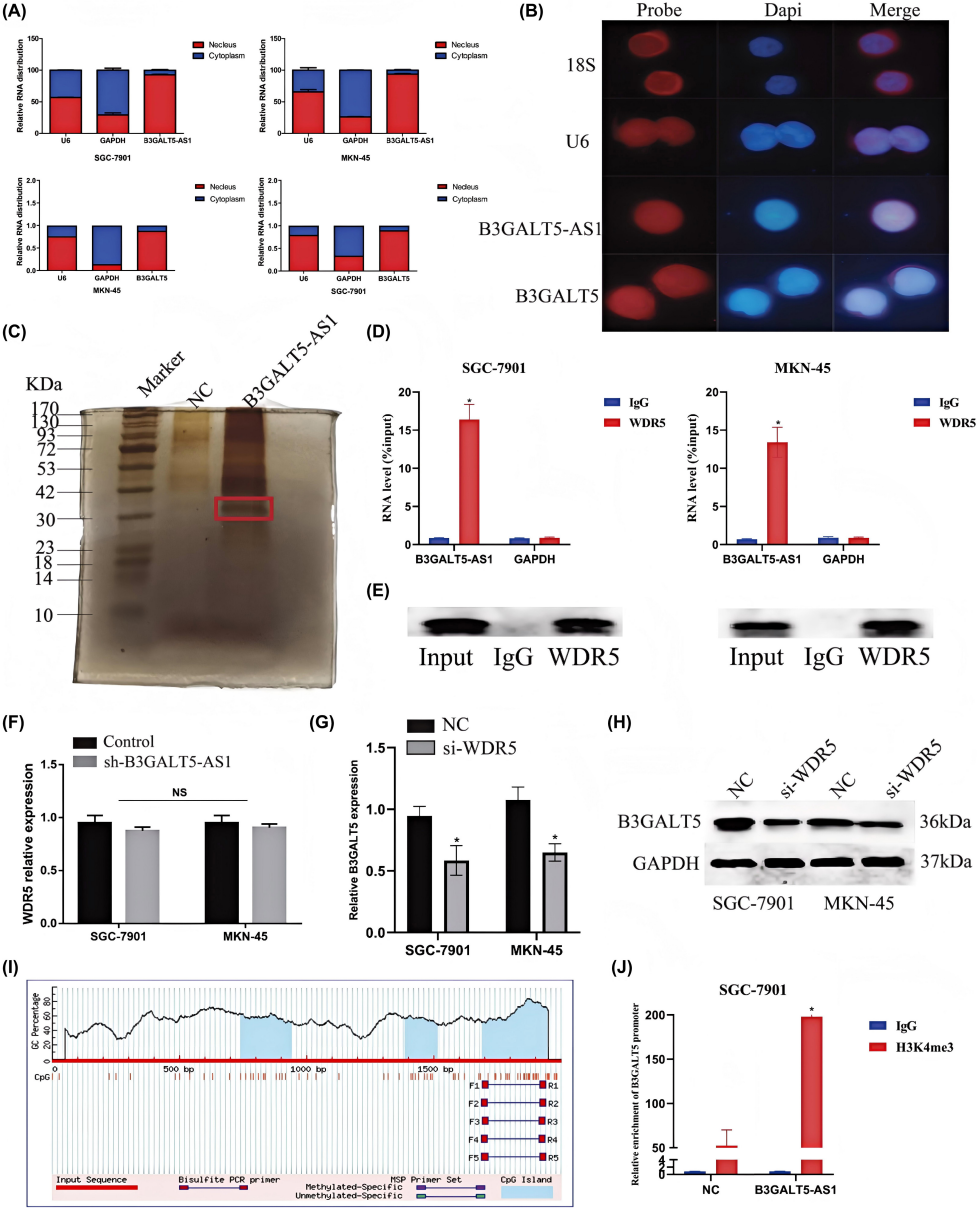
B3GALT5‐AS1 positively regulates B3GALT5 expression in GC via recruiting WDR5. (A) Nucleoplasma isolation PCR detects B3GALT5‐AS1 and B3GALT5 subcellular fractions. (B) FISH analysis was used to determine the subcellular localization of B3GALT5‐AS1 and B3GALT5. (C) Silver staining of the SDS‐PAGE gel showing the separated proteins pulled down by biotin‐labelled B3GALT5‐AS1. (D and E) RIP assay to validate the interaction between endogenous B3GALT5‐AS1 and WDR5 in SGC‐7901 and MKN‐45 cells. (F) QRT‐PCR was used to detect WDR5 expression after B3GALT5‐AS1 transfection. (G and H) qRT‐PCR and western blotting were used to detect B3GALT5 expression after si‐WDR5 transfection. (I) The presence of CpG islands in the B3GALT5 promoter region. (J) A ChIP assay was performed to detect H3K4me3 modifications of the B3GALT5 promoter. *p < 0.05, ***p < 0.001.

Furthermore, subcellular localization analysis revealed that B3GALT5‐AS1 and B3GALT5 were both enriched in the nucleus (Figure 8A,B). To determine how B3GALT5‐AS1 regulated the expression of B3GALT5, RNA pull‐down analysis was carried out. The results showed that the specific band was located between 30 and 42 kDa (Figure 8C). We then subjected gels in this range to mass spectrometry. Based on the functional annotation of mass spectrometry analysis and previous literature, WDR5 was selected for further study. WDR5 was reported to be recruited to the promoter region of its target gene by lncRNAs and to activate target gene promoter DNA demethylation to promote gene expression.^26^ It is a key component of the histone H3K4 methyltransferase complex and can catalyse H3K4me3, which is associated with the transcriptional activation of target genes.^27^ Similarly, the bioinformatics prediction revealed the presence of CpG islands in the B3GALT5 promoter region (Figure 8I). Therefore, we predicted that B3GALT5‐AS1 might influence the expression of B3GALT5 by recruiting WDR5 to its promoter region and regulating H3K4me3 levels. RIP assays performed with an anti‐WDR5 antibody also confirmed the interaction between WDR5 and B3GALT5‐AS1 (Figure 8D,E). Interestingly, qRT‐PCR showed that silencing B3GALT5‐AS1 markedly reduced the expression of B3GALT5, but had no influence on WDR5, and treatment with si‐WDR5 decreased the expression of B3GALT5 at the mRNA and protein levels (Figure 8F–H). In addition, the results of the ChIP assays showed that enforced expression of B3GALT5‐AS1 markedly increased the active modification H3K4me3 of the B3GALT5 promoter region (Figure 8J). Taken together, these data suggest that B3GALT5‐AS1 was able to promote B3GALT5 level by recruiting WDR5 to its promoter region of B3GALT5 and regulating H3K4me3 levels in its promoter region.

## 4 DISCUSSION

Recently, growing evidence has shown that lncRNAs play a crucial role in tumorigenesis in several cancers.^28^, ^29^, ^30^ LncRNA B3GALT5‐AS1 is a novel antisense lncRNA, and studies have shown that antisense lncRNAs are often expressed from cancer‐associated neighbouring genes together with concordant expression of their sense genes.^31^, ^32^ For instance, HIF‐1α induced NR2F1‐AS1 upregulation in pancreatic cancer (PC) and highly expressed NR2F1‐AS1 promoted PC cell proliferation, migration, and invasion by modulating the expression of its sense gene NR2F1, which activates the AKT/mTOR pathway in PC.^33^ Tian et al. reported that lncRNA DPP10‐AS1 is upregulated in lung cancer, and high expression of DPP10‐AS1 is associated with poor prognosis in lung cancer. Furthermore, DPP10‐AS1 promotes tumorigenesis in lung cancer. Mechanistically, DPP10‐AS1 positively modulates DPP10 expression, and hypomethylation of DPP10‐AS1 and DPP10 contributes to their coordinated upregulation in lung cancer.^34^ Jiang et al. found that the expression of MARCKSL1‐2 was significantly reduced in DTX‐resistant LAD cells. Mechanism studies have shown that MARCKSL1‐2 can recruit SUZ12 to the promoter region of HDAC1, enhancing its H3K27me3 levels and downregulating the HDAC1 levels. Low expression of HDAC1 can restore the activity of miR‐200b, thereby reducing the resistance of LAD cells to DTX.^14^ B3GALT5‐AS1 was first reported in colon cancer and was found to be downregulated in colon cancer. B3GALT5‐AS1 suppressed cell proliferation but promoted invasion and migration of colon cancer cells In addition, B3GALT5‐AS1 suppressed colon cancer liver metastasis via miR‐203/ZEB2‐SNAI2/EMT axis.^35^ Similarly, B3GALT5‐AS1 was downregulated in hepatocellular carcinoma, and B3GALT5‐AS1 inhibited tumour progression in vivo and in vitro. Furthermore, B3GALT5‐AS1 negatively regulates miR‐934 and inhibits the PI3K/AKT pathway to suppress hepatocellular carcinoma.^36^

Due to the tissue and spatial specificity of lncRNAs, their regulatory effects in different diseases are diverse. In the present study, we found that the expression of B3GALT5‐AS1 in GC was enhanced and that highly expressed B3GALT5‐AS1 was associated with lymphatic metastasis and TNM stage. At the same time, data downloaded from the TCGA database also indicated that overexpression of B3GALT5‐AS1 was related to poor prognosis. Knockdown of B3GALT5‐AS1 suppressed GC cell proliferation, invasion, migration, and angiogenesis in vitro. Simultaneously, a nude mouse transplantation tumour experiment showed that silencing B3GALT5‐AS1 could inhibit GC cell growth in vivo. Furthermore, immunohistochemical analysis showed that knocking down B3GALT5‐AS1 could significantly inhibit the proliferation of GC cells and promote mesenchymal epithelial transformation (MET). Conversely, B3GALT5‐AS1 overexpression promoted GC cell proliferation, invasion, migration, and angiogenesis. These data indicate that B3GALT5‐AS1 is critical for GC and could be used as a potential biomarker for the diagnosis of GC.

Mechanistically, B3GLAT5‐AS1 was mainly localized in the nucleus, and combined with bioinformatic analysis, B3GALT5‐AS1 could regulate B3GALT5 in GC. Evidence has shown that lncRNAs can interact with chromatin regulatory proteins to promote their recruitment to chromatin, thereby regulating transcriptional activity and influencing the expression of neighbouring genes.^13^ Xu et al. found that lncRNA SATB2‐AS1 expression is downregulated in colorectal cancer (CRC). Low expression of SATB2‐AS1 inhibits CRC metastasis and immune response. Simultaneously, SATB2‐AS1 directly combines with WDR5 and GADD45A to cis‐activate SATB2 transcription and affect SATB2 expression.^37^ Another study showed that TM4SF19‐AS1 is significantly highly expressed in lung squamous cell carcinoma (LSCC) and affects LSCC cell proliferation. Mechanistically, TM4SF19‐AS1 directly binds to WDR5, which is recruited to the TM4SF19 promoter region, and modulates DNA demethylation. Subsequently, TM4SF19‐AS1 mediated TM4SF19 expression by recruiting WDR5, thereby promoting the proliferation and adhesion of LSCC.^38^ In our study, B3GALT5‐AS1 and B3GALT5 coordinated upregulation in GC, and the expression of B3GALT5 was positively correlated with that of B3GLAT5‐AS1. We also found that B3GALT5‐AS1 could interact with WDR5, which is an important chromatin‐modulating protein, and recruited WDR5 to the B3GALT5 promoter region and modulated the methylation level of B3GALT5. Moreover, the knockdown of B3GALT5‐AS1 inhibited the expression of B3GALT5 but did not affect WDR5, whereas silencing WDR5 decreased the expression of B3GALT5. Rescue assays confirmed that the effects of si‐B3GALT5 on GC cell proliferation, invasion, and angiogenesis were partially reversed by overexpressing B3GALT5‐AS1. Studies have shown that B3GALT5 is a precursor of sialyl Lewis A (SLe^a^), also known as CA19‐9. Overexpression of the HBV X protein in Chang cells enhances SLe^a^ expression and mediates cell adhesion and metastasis to endothelial cells by inducing B3GALT5. Therefore, high expression of B3GALT5 may promote tumour metastasis through SLe^a^ in hepatocellular carcinoma tissues.^39^, ^40^ According to a previous study, B3GALT5‐AS1 regulates GC metastasis and EMT plays an important role in tumour metastasis. Western blotting showed that the expression of ZEB1, β‐catenin, and EMT‐related proteins was affected in different treatment groups.

**FIGURE 9:**
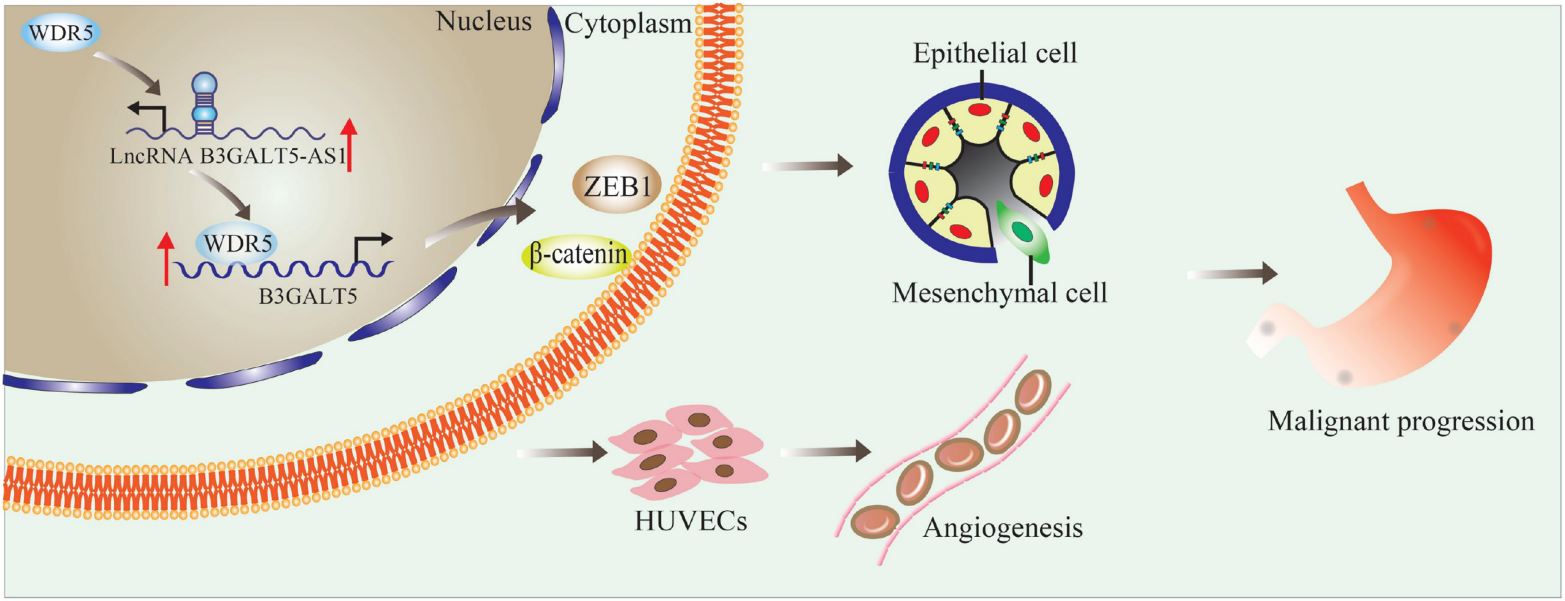
Summary of the mechanism underlying B3GALT5‐AS1 recruits WDR5 modulates B3GALT5/β‐catenin/ZEB1 pathway.

Collectively, B3GALT5‐AS1 is upregulated in GC and regulates the expression of B3GALT5 by recruiting WDR5 to the promoter region of B3GALT5. B3GALT5 enhances the expression of activated β‐catenin and ZEB1 to promote the invasion and migration of GC cells and accelerate the malignant process of GC metastasis (Figure 9).

## AUTHOR CONTRIBUTIONS

**Wei Feng:** Data curation (equal); formal analysis (equal); funding acquisition (equal). **Yelan Tang:** Data curation (equal); formal analysis (equal); investigation (equal); methodology (equal). **Rongrong Jing:** Writing – original draft (equal). **Shaoqing Ju:** Funding acquisition (equal). **Wei Zong:** Conceptualization (equal); funding acquisition (equal).

## FUNDING INFORMATION

This project was supported by the National Natural Science Foundation of China (Grant number 82102480), Jiangsu Provincial Key Medical Discipline (Laboratory) ZDXK202240, and the Postgraduate Research & Practice Innovation Program of Jiangsu Province (KYCX24_3598).

## CONFLICT OF INTEREST STATEMENT

The authors declare that there is no conflict of interest in this study.

## Supporting information


Table S1.

Figure S1.

Figure S2.

## Data Availability

The datasets used and/or analysed during the current study are available from the corresponding author upon reasonable request.

## References

[1] Siegel RL , Miller KD , Wagle NS , Jemal A . Cancer statistics, 2023. CA Cancer J Clin. 2023;73:17‐48. 36633525 10.3322/caac.21763

[2] Cao M , Li H , Sun D , Chen W . Cancer burden of major cancers in China: a need for sustainable actions. Cancer Commun. 2020;40:205‐210. 10.1002/cac2.12025 PMC7667573 32359212

[3] Necula L , Matei L , Dragu D , et al. Recent advances in gastric cancer early diagnosis. World J Gastroenterol. 2019;25:2029‐2044. 31114131 10.3748/wjg.v25.i17.2029 PMC6506585

[4] Kanda M , Kodera Y . Recent advances in the molecular diagnostics of gastric cancer. World J Gastroenterol. 2015;21:9838‐9852. 26379391 10.3748/wjg.v21.i34.9838 PMC4566379

[5] Smyth EC , Nilsson M , Grabsch HI , van Grieken NC , Lordick F . Gastric cancer. Lancet. 2020;396:635‐648. 32861308 10.1016/S0140-6736(20)31288-5

[6] Zong W , Ju S , Jing R , Cui M . Long non‐coding RNA‐mediated regulation of signaling pathways in gastric cancer. Clin Chem Lab Med. 2018;56:1828‐1837. 29804098 10.1515/cclm-2017-1139

[7] He Y , Vogelstein B , Velculescu VE , Papadopoulos N , Kinzler KW . The antisense transcriptomes of human cells. Science. 2008;322:1855‐1857. 19056939 10.1126/science.1163853 PMC2824178

[8] Shen T , Li H , Song Y , et al. Antisense transcription regulates the expression of sense gene via alternative polyadenylation. Protein Cell. 2018;9:540‐552. 29273853 10.1007/s13238-017-0497-0 PMC5966356

[9] Villegas VE , Zaphiropoulos PG . Neighboring gene regulation by antisense long non‐coding RNAs. Int J Mol Sci. 2015;16:3251‐3266. 25654223 10.3390/ijms16023251 PMC4346893

[10] Gu J , Li Y , Fan L , et al. Identification of aberrantly expressed long non‐coding RNAs in stomach adenocarcinoma. Oncotarget. 2017;8:49201‐49216. 28484081 10.18632/oncotarget.17329 PMC5564761

[11] Feng W , Zong W , Li Y , Shen X , Cui X , Ju S . Abnormally expressed long noncoding RNA B3GALT5‐AS1 may serve as a biomarker for the diagnostic and prognostic of gastric cancer. J Cell Biochem. 2020;121:557‐565. 31338903 10.1002/jcb.29296

[12] Zhou M , Guo X , Wang M , Qin R . The patterns of antisense long non‐coding RNAs regulating corresponding sense genes in human cancers. J Cancer. 2021;12:1499‐1506. 33531995 10.7150/jca.49067 PMC7847652

[13] Kopp F , Mendell JT . Functional classification and experimental dissection of long noncoding RNAs. Cell. 2018;172:393‐407. 29373828 10.1016/j.cell.2018.01.011 PMC5978744

[14] Jiang M , Qi F , Zhang K , et al. MARCKSL1‐2 reverses docetaxel‐resistance of lung adenocarcinoma cells by recruiting SUZ12 to suppress HDAC1 and elevate miR‐200b. Mol Cancer. 2022;21:150. 35864549 10.1186/s12943-022-01605-w PMC9306054

[15] Engle DD , Tiriac H , Rivera KD , et al. The glycan CA19‐9 promotes pancreatitis and pancreatic cancer in mice. Science. 2019;364:1156‐1162. 31221853 10.1126/science.aaw3145 PMC6705393

[16] Liao YM , Wang YH , Hung JT , et al. High B3GALT5 expression confers poor clinical outcome and contributes to tumor progression and metastasis in breast cancer. Breast Cancer Res. 2021;23:5. 33413566 10.1186/s13058-020-01381-9 PMC7792347

[17] Ding Y , Feng W , Ge JK , et al. Serum level of long noncoding RNA B3GALT5‐AS1 as a diagnostic biomarker of colorectal cancer. Future Oncol. 2020;16:827‐835. 32207329 10.2217/fon-2019-0820

[18] Bure IV , Nemtsova MV , Zaletaev DV . Roles of E‐cadherin and noncoding RNAs in the epithelial‐mesenchymal transition and progression in gastric cancer. Int J Mol Sci. 2019;20(12):2870. 31212809 10.3390/ijms20122870 PMC6627057

[19] Nasrollahzadeh‐Khakiani M , Emadi‐Baygi M , Schulz WA , Nikpour P . Long noncoding RNAs in gastric cancer carcinogenesis and metastasis. Brief Funct Genomics. 2017;16:129‐145. 27122631 10.1093/bfgp/elw011

[20] Feng YN , Li BY , Wang K , Li XX , Zhang L , Dong XZ . Epithelial–mesenchymal transition‐related long noncoding RNAs in gastric carcinoma. Front Mol Biosci. 2022;9:977280. 36310592 10.3389/fmolb.2022.977280 PMC9605205

[21] Dou R , Han L , Yang C , et al. Upregulation of LINC00501 by H3K27 acetylation facilitates gastric cancer metastasis through activating epithelial–mesenchymal transition and angiogenesis. Clin Transl Med. 2023;13:e1432. 37867401 10.1002/ctm2.1432 PMC10591115

[22] Kitadai Y . Angiogenesis and lymphangiogenesis of gastric cancer. J Oncol. 2010;2010:468725. 20369064 10.1155/2010/468725 PMC2847386

[23] Kalfon T , Loewenstein S , Gerstenhaber F , et al. Gastric cancer‐derived extracellular vesicles (EVs) promote angiogenesis via angiopoietin‐2. Cancer. 2022;14(12):2953. 10.3390/cancers14122953 PMC9221039 35740619

[24] Cong N , Du P , Zhang A , et al. Downregulated microRNA‐200a promotes EMT and tumor growth through the wnt/β‐catenin pathway by targeting the E‐cadherin repressors ZEB1/ZEB2 in gastric adenocarcinoma. Oncol Rep. 2013;29:1579‐1587. 23381389 10.3892/or.2013.2267

[25] Yuan K , Xie K , Lan T , et al. TXNDC12 promotes EMT and metastasis of hepatocellular carcinoma cells via activation of β‐catenin. Cell Death Differ. 2020;27:1355‐1368. 31570854 10.1038/s41418-019-0421-7 PMC7206186

[26] Ma M , Zhang Y , Weng M , et al. lncRNA GCAWKR promotes gastric cancer development by scaffolding the chromatin modification factors WDR5 and KAT2A. Mol Ther. 2018;26:2658‐2668. 30274785 10.1016/j.ymthe.2018.09.002 PMC6225079

[27] Yang R , Liu N , Li T , et al. LncRNA AC142119.1 facilitates the progression of neuroblastoma by epigenetically initiating the transcription of MYCN. J Transl Med. 2023;21:659. 37741985 10.1186/s12967-023-04535-3 PMC10518117

[28] Tan YT , Lin JF , Li T , Li JJ , Xu RH , Ju HQ . LncRNA‐mediated posttranslational modifications and reprogramming of energy metabolism in cancer. Cancer Commun. 2021;41:109‐120. 10.1002/cac2.12108 PMC7896749 33119215

[29] McCabe EM , Rasmussen TP . lncRNA involvement in cancer stem cell function and epithelial–mesenchymal transitions. Semin Cancer Biol. 2021;75:38‐48. 33346133 10.1016/j.semcancer.2020.12.012

[30] Yu Z , Tang H , Chen S , et al. Exosomal LOC85009 inhibits docetaxel resistance in lung adenocarcinoma through regulating ATG5‐induced autophagy. Drug Resist Updat. 2023;67:100915. 36641841 10.1016/j.drup.2022.100915

[31] Yuan J , Liu Z , Song R . Antisense lncRNA As‐SLC7A11 suppresses epithelial ovarian cancer progression mainly by targeting SLC7A11. Pharmazie. 2017;72:402‐407. 29441937 10.1691/ph.2017.7449

[32] Yan X , Cong B , Chen Q , et al. Silencing lncRNA HOXA10‐AS decreases cell proliferation of oral cancer and HOXA10‐antisense RNA can serve as a novel prognostic predictor. J Int Med Res. 2020;48:300060520934254. 32776855 10.1177/0300060520934254 PMC7418258

[33] Liu Y , Chen S , Cai K , et al. Hypoxia‐induced long noncoding RNA NR2F1‐AS1 maintains pancreatic cancer proliferation, migration, and invasion by activating the NR2F1/AKT/mTOR axis. Cell Death Dis. 2022;13:232. 35283481 10.1038/s41419-022-04669-0 PMC8918554

[34] Tian H , Pan J , Fang S , et al. LncRNA DPP10‐AS1 promotes malignant processes through epigenetically activating its cognate gene DPP10 and predicts poor prognosis in lung cancer patients. Cancer Biol Med. 2021;18:675‐692. 34106559 10.20892/j.issn.2095-3941.2020.0136 PMC8330531

[35] Wang L , Wei Z , Wu K , et al. Long noncoding RNA B3GALT5‐AS1 suppresses colon cancer liver metastasis via repressing microRNA‐203. Aging. 2018;10:3662‐3682. 30530918 10.18632/aging.101628 PMC6326654

[36] Chen E , Zhou B , Bian S , Ni W , Chen Z . The lncRNA B3GALT5‐AS1 functions as an HCC suppressor by regulating the miR‐934/UFM1 axis. J Oncol. 2021;2021:1776432. 34721576 10.1155/2021/1776432 PMC8550832

[37] Xu M , Xu X , Pan B , et al. LncRNA SATB2‐AS1 inhibits tumor metastasis and affects the tumor immune cell microenvironment in colorectal cancer by regulating SATB2. Mol Cancer. 2019;18:135. 31492160 10.1186/s12943-019-1063-6 PMC6729021

[38] Luo M , Xie L , Su Y , et al. TM4SF19‐AS1 facilitates the proliferation of lung squamous cell carcinoma by recruiting WDR5 to mediate TM4SF19. Mol Cell Probes. 2022;65:101849. 35987447 10.1016/j.mcp.2022.101849

[39] Zhang X , Liu H , Wang H , et al. B3galt5 deficiency attenuates hepatocellular carcinoma by suppressing mTOR/p70s6k‐mediated glycolysis. Cell Mol Life Sci. 2022;80:8. 36495345 10.1007/s00018-022-04601-x PMC11072394

[40] Mare L , Caretti A , Albertini R , Trinchera M . CA19.9 antigen circulating in the serum of colon cancer patients: where is it from? Int J Biochem Cell Biol. 2013;45:792‐797. 23333853 10.1016/j.biocel.2013.01.004

