# Supplementary material for: Highly expressed B3GALT5‐AS1 contributes to gastric cancer progression by recruiting WDR5 to mediate B3GALT5 and regulating β‐catenin/ZEB1 axis
Source: J Cell Mol Med. 2024 Sep 3;28(17):e70061. doi: 10.1111/jcmm.70061 (PMC11369489; doi:10.1111/jcmm.70061)
Supplement: Supplementary file 1 — Table S1. [file JCMM-28-e70061-s003.docx]

**Supplementary Table S1**

**Primers used for qRT-qPCR.**

| **Primers** | **Sequence** |
| --- | --- |
| 18SrRNA | **F:** 5’- GTAACCCGTTGAACCCCATT -3’ |
|  | **R:** 5’- CCATCCAATCGGTAGTAGCG -3’ |
| B3GALT5-AS1 | **F:** 5’- GATCCACGTCCAGGCTCACT -3’ |
|  | **R:** 5’- GTGCTGGCTGTCAGGATGAG -3’ |
| B3GALT5 | **F:** 5’- GCAGATCTATGGCTTTCCCGAAGATG -3’ |
|  | **R:** 5’- GTCTCGAGTCAGACAGGCGGACAAT -3’ |
| WDR5 | **F:** 5’- TGCTGCAACTTCAATCCCCA -3’ |
|  | **R:** 5’- GTGTCCCAGATGCGACAGAG -3’ |

**Supplementary Table S2**

**Information for antibodies**

| **Name** | **Target Sequence (5' to 3')** |
| --- | --- |
| **shRNA** | |
| sh1-B3GALT5-AS1 | GGAGAACCGTCCCAAAGTTAA |
| Sh2-B3GALT5-AS1 | GGGACCATTATTCTGCCAACT |
| **siRNA** | |
| si-WDR5 | TCTGGAACCTTCAGACGAATT |
| si-B3GALT5 | GCAAGTGGTTTGTCAGTAATT |

**Supplementary Table S3**

**Information for antibodies**

|  |  | **Western blot** | **ChIP** | **RIP** |
| --- | --- | --- | --- | --- |
| B3ALT5 Rabbit Polyclonal | Absin | 1:500 |  |  |
| WDR5 (D9E1I) Rabbit mAb | cell signaling technology #13105 | 1:1000 |  | 1:50 |
| Tri-Methyl-Histone H3 (Lys4) (C42D8) Rabbit mAb | cell signaling technology #9751 |  | 1:50 |  |
| GAPDH Rabbit Polyclonal | Abcam #ab181602 | 1:10000 |  |  |
| Vimentin Rabbit Polyclonal | Abcam #ab8069 | 1:1000 |  |  |
| E-cadherin Rabbit Polyclonal | Abcam #ab231303 | 1:1000 |  |  |
| ZEB1 Rabbit Polyclonal | proteintech #10372-2-AP | 1:800 |  |  |
| β-cantenin | proteintech #51067-2-AP | 1:5000 |  |  |
