# Supplementary figures and images for: Highly expressed B3GALT5‐AS1 contributes to gastric cancer progression by recruiting WDR5 to mediate B3GALT5 and regulating β‐catenin/ZEB1 axis
Source: J Cell Mol Med. 2024 Sep 3;28(17):e70061. doi: 10.1111/jcmm.70061 (PMC11369489; doi:10.1111/jcmm.70061)

A

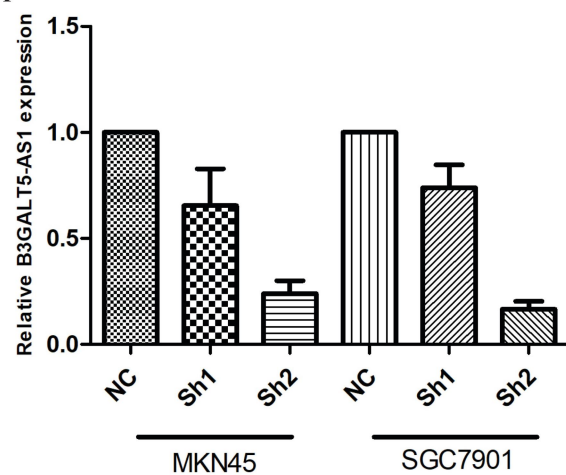

B

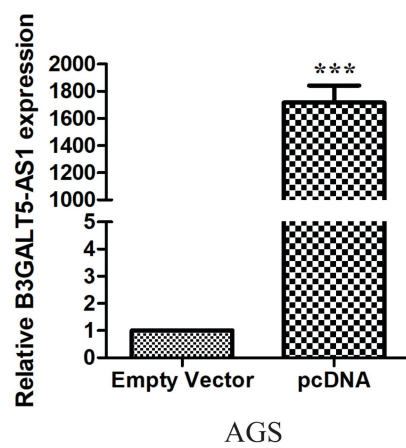

Supplement: Supplementary file 2 — Figure S1. [file JCMM-28-e70061-s002.pdf]

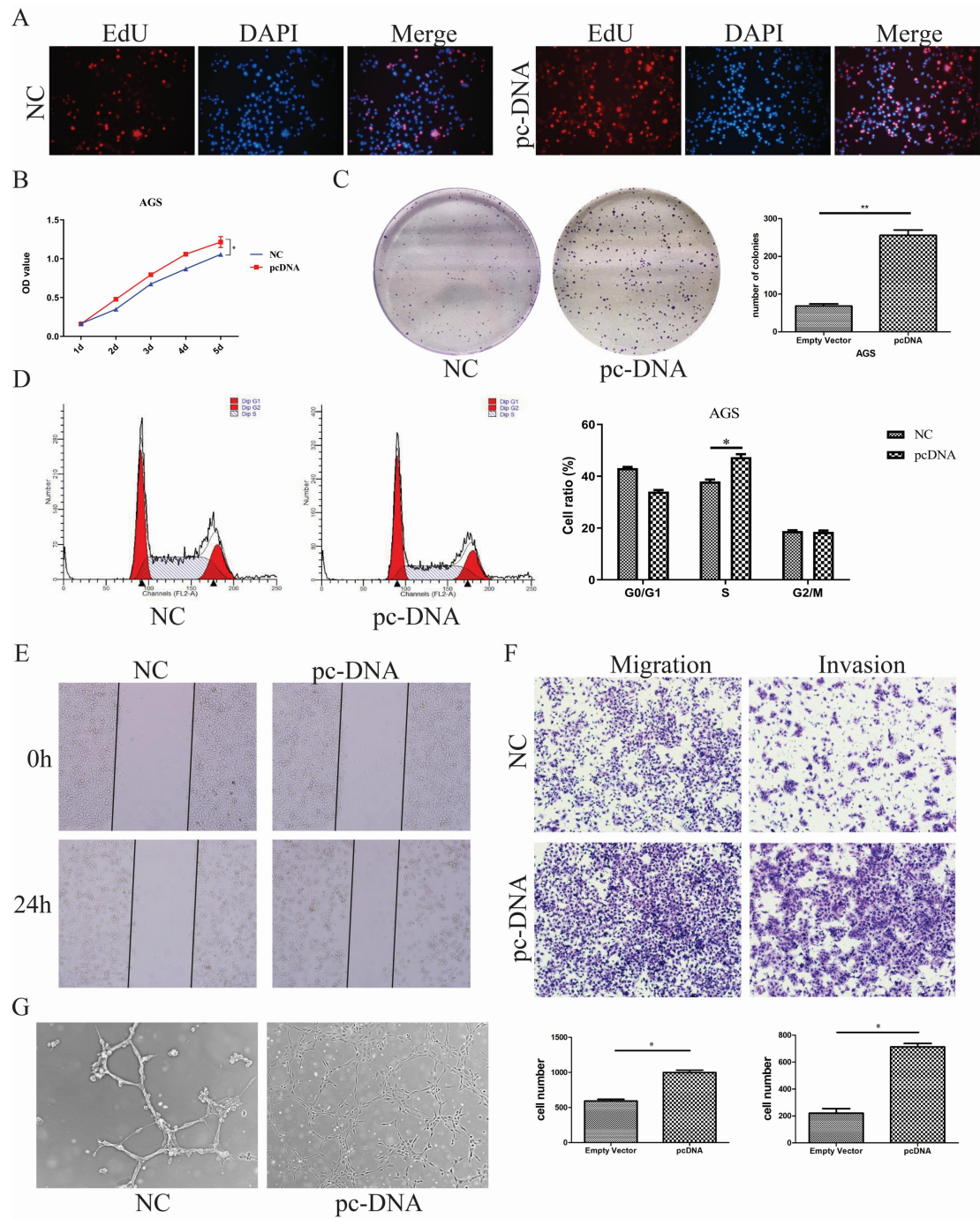

Supplement: Supplementary file 3 — Figure S2. [file JCMM-28-e70061-s001.pdf]
